# Exploring Healthy Eating Perceptions, Barriers, and Facilitators among Urban Indigenous Peoples in Saskatchewan

**DOI:** 10.3390/nu16132006

**Published:** 2024-06-25

**Authors:** Mojtaba Shafiee, Samer Al-Bazz, Ginny Lane, Michael Szafron, Hassan Vatanparast

**Affiliations:** 1 College of Pharmacy and Nutrition, University of Saskatchewan, Saskatoon, SK S7N 5A9, Canada; mojtaba.shafiee@usask.ca (M.S.); saa331@mail.usask.ca (S.A.-B.); 2 Margaret Ritchie School of Family and Consumer Sciences, University of Idaho, Moscow, ID 83843, USA; vlane@uidaho.edu; 3 School of Public Health, University of Saskatchewan, Saskatoon, SK S7N 2Z4, Canada; michael.szafron@usask.ca

**Keywords:** urban indigenous peoples, healthy eating perceptions, healthy eating barriers, healthy eating facilitators, traditional foods, Canada

## Abstract

Urban Indigenous populations encounter distinctive challenges in maintaining traditional dietary practices, compounded by the complexities of socio-economic and environmental factors and the modern urban lifestyle. This qualitative study explores the perceptions of healthy eating, along with the facilitators and barriers to such practices, among urban Indigenous peoples in Saskatoon, Regina, and Prince Albert. Through virtual interviews, we engage 14 participants from these cities. Utilizing NVivo for thematic coding, we apply inductive thematic analysis to reveal relevant themes. The study highlights a preference for nutrient-rich, natural, and minimally processed foods, with a significant emphasis on incorporating traditional Indigenous foods into diets. These preferences are deeply entwined with cultural identity and underscore the importance of traditional foods in maintaining cultural heritage and promoting well-being. Despite the intrinsic value of these traditional foods, participants face several barriers to healthy eating, including economic constraints, limited access to traditional foods, and the psychological impacts of historical trauma. Nevertheless, facilitators such as community and family support, engagement in traditional food practices, and a growing awareness of nutritional knowledge are identified as being crucial in supporting healthy dietary choices. This research underscores the complex interplay of cultural, economic, and environmental factors in shaping the dietary practices of urban Indigenous peoples.

## 1. Introduction

Indigenous peoples in Canada, comprised of First Nations, Métis, and Inuit people, represent a significant and growing segment of the nation’s population [1]. The 2021 census revealed that the Indigenous population surpassed 1.8 million, making up 5% of Canada’s overall population and marking a 9.4% rise since 2016 [1]. Furthermore, a notable shift toward urban living has been observed, with 1,090,240 Indigenous people now residing in urban areas—a growth of 11.5% from 2016 [2]. Saskatchewan emerges as a key province in this context, being home to 10.4% of Canada’s Indigenous population [3]. A considerable segment of this population is found in urban centers such as Saskatoon (15.9% or 29,880 individuals), Regina (12.4% or 23,285 individuals), and Prince Albert (8.6% or 16,125 individuals) [3]. This concentration in urban centers provides a setting for a dynamic interplay between urban Indigenous communities’ resilience, cultural richness, and the challenges they face in connecting with traditional ways of life within a modern urban setting.

The growing urban Indigenous population in Canada faces significant health disparities, including higher rates of chronic diseases like obesity, diabetes, and cardiovascular diseases [4,5]. A key factor contributing to these health issues is diet, which is greatly influenced by rapid lifestyle changes and marginalization [6,7]. This marginalization, characterized by disruptions in social and cultural norms and a decline in traditional ways of life, stems from the lasting impacts of colonization, residential schools, and systemic inequalities [8,9,10]. Government-imposed policies and resulting practices have led to substantial alterations to Indigenous life, notably affecting traditional food-gathering practices and overall health [7,11]. The adoption of a Western lifestyle has resulted in the diets of Indigenous people being high in calories but low in nutritional value [12,13,14]. Our previous research demonstrated that the dominant dietary pattern among off-reserve Indigenous adults in Canada in 2015 was identified as being “Unhealthy”, characterized by low nutritional quality as reflected by low Nutrient-Rich Food (NRF) scores [12]. This is contrary to Indigenous peoples’ traditionally distinct food systems and dietary customs, which are deeply entrenched in their cultural traditions and environmental settings [15,16]. Our recent study further explored the dietary preferences of urban Indigenous individuals in Saskatchewan, showing a notable tendency towards a blend of store-bought and traditional foods [17]. Specifically, 41.2% preferred mostly store-bought foods with some traditional components, while 21.7% maintained a balance between traditional and store-bought foods [17]. This diversity in dietary preferences underscores the importance of exploring urban Indigenous communities’ perceptions of healthy eating. Understanding these perceptions can illuminate cultural and personal definitions of what it means to eat healthily, which may differ significantly from mainstream dietary guidelines. This nuanced perspective was evident in a study conducted in the semi-remote community of Eeyou Istchee, where two distinct versions of “healthy eating” were identified [18]. Many community members associated healthy eating with traditional foods and their preparation methods, underscoring the value placed on ancestral dietary practices. Other community members described a medicalized interpretation of healthy eating, influenced by diagnoses and conditions that align with “southern” food access and preparation methods [18]. Investigating these views is crucial for developing culturally appropriate strategies to promote healthy eating among Indigenous populations.

In our comprehensive literature review, we highlighted that all aspects of food security—availability, accessibility, utilization, and stability—are impacted among Indigenous communities across Canada, both on reserves and in urban settings [19]. Before the COVID-19 pandemic, food insecurity was a significant concern, with 28.2% of off-reserve Indigenous households experiencing challenges in accessing sufficient and nutritious food due to financial constraints [20]. The pandemic further intensified these disparities, with our recent study revealing that over 68% of urban Indigenous peoples experienced food insecurity during the pandemic’s initial months [17], which underscored the fragile state of food access within these communities. For comparison, during the second wave of the COVID-19 pandemic in fall 2020, about 9.6% of Canadians aged 12 and older living in the 10 provinces reported experiencing food insecurity in their household within the prior 12 months [21]. These disproportionately elevated rates of food insecurity among Indigenous populations can severely affect healthy eating patterns [22,23], underlining a critical and immediate need to address the underlying factors affecting Indigenous peoples’ access to, and consumption of, healthy foods. Investigating the barriers and facilitators to healthy eating within these communities is not only crucial for developing targeted interventions that can mitigate these disparities but also for empowering Indigenous peoples to reclaim and adapt their traditional food systems and practices in the context of modern urban settings. For instance, when we inquired about the barriers to consuming traditional foods, the lack of time for hunting, fishing, foraging, or preparation emerged as the most common hurdle (54.2%), followed by issues of access/availability (37.5%), and a loss of traditional knowledge or means to cook, prepare, or obtain these foods (37.5%) [17]. Furthermore, in exploring coping mechanisms for food access challenges, reliance on community resources and government food programs was the predominant strategy (40.7%) along with adjustments to eating habits (33.3%) and support from family and social networks (27.8%) [17]. Understanding these challenges and opportunities is vital for designing policies and programs that are effective, culturally respectful, and supportive of Indigenous peoples’ rights to food sovereignty and health equity.

To the best of our knowledge, there is a gap in the literature concerning healthy eating perceptions, barriers, and facilitators among urban Indigenous peoples in Saskatchewan. Our study seeks to address this gap by investigating the perceptions, barriers, and facilitators of healthy eating among this population. Our focus is not solely on addressing the critical issue of food insecurity but also on understanding the complex interplay of cultural, economic, and urban environmental factors that influence dietary choices among urban Indigenous populations. This approach recognizes the importance of cultural relevance in dietary interventions and seeks to uncover pathways supporting the revitalization of traditional food practices alongside promoting healthy eating patterns. Additionally, our research examines how the COVID-19 pandemic has influenced the eating habits of urban Indigenous peoples. By exploring these dimensions, this study aims to contribute to the development of culturally sensitive, effective strategies to enhance food security and nutritional health within these communities.

## 2. Materials and Methods

### 2.1. Study Design

We conducted a qualitative study employing an inductive thematic analytical approach [24]. An inductive approach allowed us to explore the patterns and themes emerging directly from the data rather than relying on predetermined hypotheses to guide our analysis [25]. This methodology aligned with our objective to uncover insights grounded in the participants’ experiences and perspectives. To enhance our analysis, with the intersectionality lens, we used the Social Ecological Model (SEM) as a theoretical framework [26], which allowed us to examine the multiple levels of influence on healthy eating behaviors, ranging from individual factors to broader societal influences. The intersectionality lens allowed us to consider how overlapping social identities, such as being urban Indigenous, interact to shape the participants’ experiences and perspectives on healthy eating. Ethical approval for the study was granted by the Behavioural Research Ethics Board (Beh-REB) of the University of Saskatchewan, which ensured adherence to ethical guidelines and the protection of participants’ rights and well-being (Approval ID: Beh 3620). This study is reported in accordance with the Consolidated Criteria for Reporting Qualitative Research (COREQ) checklist [27].

### 2.2. Participant Recruitment and Sampling Strategies

The study was conducted between September and October 2023 in three major cities: Saskatoon, Regina, and Prince Albert, located in Saskatchewan, Canada. Participants were recruited through a collaborative effort with our Indigenous partner organization, the Saskatchewan Network Environments for Indigenous Health Research (SK-NEIHR), and the Saskatoon Food Bank & Learning Centre. A recruitment message and a poster were disseminated via email and subsequently shared on the SK-NEIHR’s social media platforms. Additionally, the recruitment posters were displayed at the Saskatoon Food Bank & Learning Centre, and interested participants were instructed to reach out to the provided email. The inclusion criteria for participants were as follows: being aged 18 years or older, self-identifying as an Indigenous individual, and residing in Saskatoon, Regina, or Prince Albert.

To ensure a representative sample of Indigenous perspectives within these urban environments, we employed a purposive sampling strategy. This approach allowed us to selectively recruit diverse participants that identified as First Nations or Metis, were located in one of three major Saskatchewan cities and included a range of ages and genders. Furthermore, we utilized snowball sampling as an additional recruitment method, encouraging our initial participants to spread the word about our research within their communities. This strategy helped to extend our reach within the target population, facilitating the recruitment of participants who might not have been accessible through direct outreach alone.

### 2.3. Data Collection

We used in-depth, semi-structured interviews to explore the experiences and perspectives of urban Indigenous adults. The interviews were primarily conducted using Zoom (Zoom Video Communications, San Jose, CA, USA), an online video conferencing platform, to facilitate accessibility and convenience for participants. We also offered the option of face-to-face interviews for those who preferred a more personal interaction. All interviews were conducted by a single researcher, M.S., a male PhD candidate with a background in food security and Indigenous health. As an immigrant to Canada, and belonging to a cultural group considered an ethnic minority, M.S. brought a unique perspective that enhanced his understanding and sensitivity toward the participants’ experiences. He had experience and training in qualitative research methods and thematic analysis. There was no prior relationship between the interviewer and the participants before the study, reducing bias in the data collection process. To further mitigate the potential influence of his social standing and outsider status, M.S. engaged in reflective practices throughout the research process. He consciously addressed these dynamics by fostering an open and respectful communication environment, actively listening to participants’ stories and perspectives without imposing his own preconceptions.

Participants were informed about the research team and the study’s objectives in advance, with consent forms being distributed via email at least one day prior to the scheduled interview. Interview scheduling was participant-driven, and we asked for their preferred date and time to conduct the interview, ensuring that their convenience and availability were prioritized. Each interview was conducted once, with no repeat sessions. At the beginning of each interview, the interviewer introduced himself, shared his academic and research background, and explained the purpose of the study. The aim of the introduction was to create a comfortable environment for the participants to share their experiences. The semi-structured format of the interviews allowed for a fluid conversation, providing the flexibility to explore spontaneous topics while still covering the guided questions. This approach facilitated a deeper understanding of the participants’ views and experiences. The key questions guiding our interviews were:

1. How do you define healthy eating? What do you think healthy food is?
2. In your view, what are the barriers to healthy eating for Indigenous peoples in urban areas?
3. What do you think are the facilitators to healthy eating among Indigenous peoples living in urban areas?
4. In what ways has the COVID-19 pandemic influenced your or your household’s eating habits?

Interviews were conducted until data saturation was achieved, meaning that additional interviews were not adding any new concepts that addressed the research objectives [28]. Data were securely stored in a password-protected folder on DataStore, a secured University of Saskatchewan file storage system.

### 2.4. Data Analysis

All interviews were audio-recorded via Zoom and transcribed verbatim to ensure accuracy in both data capture and analysis. The interviewer, M.S., took notes during each interview and proceeded to transcribe the recordings immediately afterward. To enhance the credibility of the data analysis, the transcribed interviews were returned to the participants for review. This member checking process allowed participants to verify the accuracy of the transcripts and confirm that their experiences and perspectives were accurately represented [29]. Participants were asked to return any revisions to the transcripts within one week.

Data were analyzed using inductive thematic analysis. Initially, open coding was conducted by the first author (M.S.) to identify sentences that were related to the research questions. Subsequently, the first and second authors (M.S. and S.A.) discussed the coding system and reconciled initial differences through re-coding with reference to the research questions. This collaborative process led to the construction of subthemes. Any similarities and differences identified during this stage were discussed with a third researcher, H.V. (last author). The final themes were determined by combining the subthemes. Theoretical saturation was achieved when the categories became dense and no new open codes could be identified from the data. NVivo version 12 was employed throughout this process. The use of NVivo facilitated an organized and systematic examination of the data and enabled the identification of key themes, patterns, and insights relevant to the study’s objectives. A word cloud was created using https://wordart.com/ to visually represent the most frequently mentioned words in participant interviews regarding their perceptions, barriers, and facilitators to healthy eating.

## 3. Results

This study included the perspectives of 14 urban Indigenous individuals: 7 from Saskatoon, 5 from Regina, and 2 from Prince Albert. Participant characteristics are detailed in Table 1. In terms of Indigenous identity, the majority (12 participants) identified as First Nations, one identified as Métis, and one did not disclose their Indigenous identity. The average age of the participants was 39.8 years, with a standard deviation of 11.7 years, and ages ranged from 21 to 61 years. The average annual household income was CAN 72,600 (SD = CAN 30,692), with a range from CAN 20,000 to CAN 120,000. The gender distribution showed a predominance of females, with 10 female and 4 male participants. Educational levels varied: eight had completed undergraduate degrees, three had high school diplomas, one had a graduate degree, and others had non-degree or professional certifications. Regarding employment, eight participants were employed, and six were unemployed. Household compositions also varied, with the majority of participants (28.6%) living in two-person households. Housing situations were predominantly rental-based, with 11 participants renting their homes, two owning their homes, and one remaining undisclosed. The duration of the interviews ranged from 20 to 49 min, with an average length of 29 min. Data saturation was achieved after the twelfth interview. To ensure thoroughness, two additional interviews were conducted; however, these did not yield any new insights, confirming that data saturation had been reached.

### 3.1. Perception of Healthy Eating

Eight major themes emerged from the thematic analysis related to perceptions of healthy eating: (1) Focus on Nutrient-Rich Foods; (2) Natural and Unprocessed Food Choices; (3) Minimization of Unhealthy Foods and Ingredients; (4) Emphasis on Cultural and Traditional Foods; (5) Home-Cooked Meals and Cooking Practices; (6) Adherence to Dietary Guidelines; (7) Balanced and Diverse Diet; and (8) Health-Adjusted Diet (Table 2).

#### 3.1.1. Theme 1: Focus on Nutrient-Rich Foods

This theme emphasizes the importance of incorporating foods that are dense in nutrients into one’s diet. It highlights a strong preference for fruits and vegetables, lean proteins, and foods that offer a high nutritional value, underscoring the belief that these food groups form the cornerstone of healthy eating.

##### Fruits and Vegetables

A significant emphasis is placed on consuming a wide variety of fruits and vegetables, recognized for their essential vitamins, minerals, and fiber:

“*Healthy eating is a ton of veggies I cook a lot with lentils and beans.*”

—Participant 1, Female

“*I define healthy eating as food that nourishes your body, like fruits, vegetables, and foods that are not processed.*”

—Participant 7, Female

##### Lean Proteins and Fish

The preference for lean meats, poultry, and fish is noted as part of a nutrient-rich diet, with participants pointing out these protein sources as essential for health:

“*Having a lot of fish in the diet, too, is helpful.*”

—Participant 4, Female

“*Healthy food…would be like vegetables, lean meats and fruits.*”

—Participant 6, Female

“*Other than that, white meat, not dark meat, fish is really healthy for you.*”

—Participant 8, Female

The selection of lean proteins and fish is framed within the broader context of seeking foods that provide essential nutrients without the adverse effects associated with higher fat content.

##### Nutrient-Dense Foods

Participants also express a focus on selecting foods that are nutrient-dense, indicating a preference for foods that provide a high level of nutrients relative to their calorie content:

“*Healthy food is…food that is nutrient-dense, and low in saturated fats.*”

—Participant 9, Male

“*Healthy food is defined as something that has nutritional value*”

—Participant 14, Male

#### 3.1.2. Theme 2: Natural and Unprocessed Food Choices

This theme centers on the prioritization of whole, natural foods over processed options, highlighting a shared belief among participants that health is significantly influenced by the quality and origin of the foods consumed.

##### Avoidance of Processed Foods and Fast Foods

Participants express a deliberate effort to avoid processed and fast foods, citing health concerns associated with these types of food. This avoidance is based on the understanding that processed foods often contain high levels of additives, sugars, and unhealthy fats that can contribute to various health issues:

“*If you eat out a lot that’s ton of sugar, ton of salt, it’s not good for you. High blood pressure, right? And so scaling down on eating fast foods is really important.*”

—Participant 1, Female

“*I stay away from fast food for sure and the processed food, like the frozen pizzas, or the frozen lunches.*”

—Participant 12, Male

##### Preference for Fresh and Natural Foods

In contrast to the foods they avoid, participants express a strong preference for fresh and natural foods. This preference is articulated as a desire for foods that have not been artificially produced or heavily processed:

“*Healthy food is eating things that are natural. So nothing that’s artificially produced in a laboratory or anything that is going to be packaged and processed in a factory.*”

—Participant 2, Female

“*Healthy food…is basically natural foods that are grown and… didn’t go through a lot of processing.*”

—Participant 10, Female

#### 3.1.3. Theme 3: Minimization of Unhealthy Foods and Ingredients

This theme reflects a conscious effort among participants to avoid or reduce the intake of foods and ingredients considered detrimental to health. It captures a broad range of dietary adjustments focused on limiting sugar, salt, certain carbohydrates, red meat, and processed foods.

##### Reduction in Sugar and Salt

Participants emphasize the importance of reducing sugar and salt in their diets, recognizing the health risks associated with high consumption of these ingredients:

“*I try to stay away from white bread, sugar, starches of any type…*”

—Participant 1, Female

This sentiment is echoed in the avoidance of processed foods, often high in added sugars and salt:

“*…and staying away from processed foods, sugar, salt, starch.*”

—Participant 1, Female

##### Limited Intake of Starches and Carbs

The limited intake of starches and carbohydrates is another aspect through which participants express their perception of healthy eating:

“*…healthy eating would be…not so much carbs or starches, or stuff like that…because carbs turn to sugar.*”

—Participant 8, Female

##### Limited Intake of Red Meat

Participants also perceive the moderate intake of red meat consumption as a component of healthy eating:

“*White meat, not dark meat, fish is really healthy for you. Basically, those would be the most healthy things for a person.*”

—Participant 8, Female

“*And then, it’s mostly vegetables and meat. If I had to choose food that I would eat constantly, it would just be meat—less red meat, but more so meat and vegetables like legumes, and stuff like that.*”

—Participant 13, Male

##### Limited Intake of Junk Food

Avoiding junk food emerges as a clear perception of what is necessary for a healthy diet:

“*Healthy food in my opinion is fruits and vegetables, low carbohydrates and no junk food.*”

—Participant 7, Female

“*We like to have wild meat in my diet and not too much junk food.*”

—Participant 14, Male

#### 3.1.4. Theme 4: Emphasis on Cultural and Traditional Foods

This theme highlights the participants’ strong belief in the health benefits of foods that are part of their cultural heritage and traditional dietary practices. They value natural, unprocessed foods that have nourished their communities for many generations. This reflects a deep connection between their cultural identity and their views on healthy eating.

Participants strongly associate healthy eating with a return to Indigenous foods, emphasizing the nutritional and cultural importance of consuming traditional foods like fish, berries, moose meat, rabbit, and deer:

“*Healthy eating is going back to those Indigenous foods. A lot of fish, berries, …and moose meat. …. Rabbit, deer, …all these foods that are Indigenous to us.*”

—Participant 1, Female

This theme also covers the broader practice of foraging and sourcing food locally, underscoring the value placed on food that comes directly from the land, as follows:

“*…a lot of foods that come from the land, so anything that can be sourced locally… your fruits, your vegetables, your grains, your local meat sources, and dairy from local farmers and producers*”

—Participant 2, Female

#### 3.1.5. Theme 5: Home-Cooked Meals and Cooking Practices

This theme highlights a crucial aspect of participants’ perceptions of healthy eating, emphasizing the significant role that preparing meals at home plays in maintaining a nutritious diet. It underscores the belief that cooking one’s own food is not only a practical approach to controlling ingredients and ensuring dietary quality but also an essential practice for achieving overall health and well-being.

Participants emphasize the importance of minimizing fast food consumption in favor of home-cooked meals:

“*Scaling down on eating fast foods is really important and making your own food.*”

—Participant 1, Female

“*A lot of healthy eating requires for you to cook your own food. If you eat out a lot that’s ton of sugar, ton of salt, it’s not good for you.*”

—Participant 1, Female

Furthermore, the practice of meal prepping is highlighted as a practical strategy for ensuring consistent access to nutritious meals throughout the week:

“*So every Sunday I make a ton of food for the week. Right? So I do all my veggie prep and I make my meals ahead of time that way I’m not stopping to eat … a piece of pizza or whatever for lunch.*”

—Participant 1, Female

#### 3.1.6. Theme 6: Adherence to Dietary Guidelines

This theme shows participants’ dependence on recognized nutritional guidelines to shape their healthy eating habits. This adherence underscores a trust in official dietary recommendations, such as those provided by the Canada Food Guide, as essential tools for guiding food choices and ensuring a balanced diet. However, it is important to consider how this reliance on Western scientific guidance might supplant traditional knowledge and dietary practices. The integration of these guidelines into everyday eating habits might overlook Indigenous food traditions and knowledge systems that have supported communities for generations. Participants often refer to the Canada Food Guide as their main guide for healthy eating, indicating a widespread belief that adhering to these guidelines equates to practicing healthy eating:

“*…I define healthy eating…from the 4 Canadian food groups. …dairy, meat, and so. That’s been around forever. …if we’re eating healthy, we incorporate in our meals a little bit of every section.*”

—Participant 10, Female

“*Healthy eating is getting your vegetables, and eating in accordance with the Canadian Food Guide, …We have been taught that in school. And even my kids have been taught that. You can’t just go with one food. It’s best for the body to have each of the food groups, with dairy and meat.*”

—Participant 12, Male

#### 3.1.7. Theme 7: Balanced and Diverse Diet

This theme highlights the perception among participants that healthy eating is fundamentally about achieving balance and diversity in one’s diet. It underscores the importance of incorporating a variety of food groups and practicing moderation to maintain health and well-being.

##### Well-Balanced Diet

Participants consistently emphasize the significance of a well-balanced diet that includes a wide range of food groups:

“*Healthy eating… is eating a well-balanced diet that includes a variety of food groups and foods from all different sources.*”

—Participant 2, Female

“*Healthy eating would be eating… a balanced diet. …incorporate all the food groups.*”

—Participant 4, Female

##### Moderation and Inclusion

A key element to maintaining a balanced and diverse diet, as expressed by participants, is the principle of moderation and inclusion. They view these as crucial to their understanding of healthy eating:

“*Healthy eating is eating everything in moderation. …a variety of foods, so it’s not cutting out like sweets or desserts, but eating everything in moderation.*”

—Participant 2, Female

#### 3.1.8. Theme 8: Health-Adjusted Diet

This theme underscores the nuanced approach participants take towards healthy eating, highlighting how individual health conditions and sensitivities significantly influence dietary choices. It reveals an understanding that healthy eating is not a universal solution but needs to be customized to address the individual health requirements and nutritional needs of each person.

Participants spoke about adjusting their diets to manage and accommodate various health conditions:

“*I have* [a chronic health condition]. *So that affects my intake and what I can and cannot eat.*”

—Participant 3, Female

Similarly, food sensitivities and allergies play a significant role in shaping participants’ dietary choices:

“*But I can’t eat bread because I get bloated. I’m pretty sure I’m* [a chronic health condition]. *So that’s affected my choices for food a lot.*”

—Participant 13, Male

Moreover, participants discussed finding suitable alternatives to meet their nutritional needs despite having dietary restrictions:

“*Healthy food is food that is good for your body. Like, I am lactose intolerant, so I’ll try to get my dairy from lactose-free yogurt, and also from cheese that’s lactose-intolerant-friendly.*”

—Participant 14, Male

**Figure 1:**
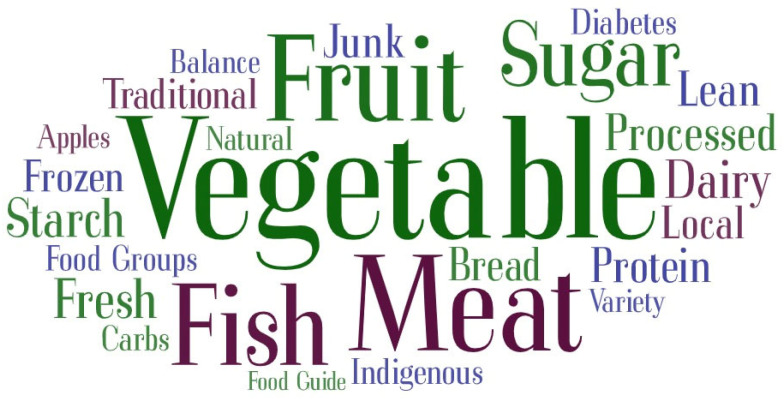
Word cloud of frequently used words in interviews on perception of healthy eating.

The word cloud for the most frequently used words in the interviews on perception of healthy eating is shown in Figure 1.

### 3.2. Barriers to Healthy Eating

Nine major themes emerged from the thematic analysis of barriers to healthy eating: (1) Economic Constraints and Poverty; (2) Access and Availability Issues; (3) Lack of Nutrition Education and Awareness; (4) Urban Lifestyle and Technological Influences; (5) Governmental and Policy Barriers; (6) Cultural and Community Factors; (7) Psychological Effects of Historical Trauma; (8) Climate Change and Seasonal Variations; and (9) Personal Dietary Preferences and Attitudes (Table 3).

#### 3.2.1. Theme 1: Economic Constraints and Poverty

This theme highlights how financial limitations and socioeconomic status significantly restrict the ability to maintain a healthy diet. It illustrates the complex relationship between economic stability and access to nutritious foods, highlighting how financial challenges impact healthy eating choices.

##### Cost and Affordability of Healthy Foods

Participants frequently mention the high cost of healthy, organic, and fresh produce as a major barrier to maintaining a nutritious diet:

“*Inflation rates and the price of food… As we go to the grocery stores or even farmers markets, a lot of the prices for healthy and organic and fresh produce is … through the roof.*”

—Participant 2, Female

“*Most people aren’t gonna read the labels when you have the bare minimum of money to spend on groceries. You’re not going to read labels if you have like, only a hundred dollars or $60 to buy groceries, and you have to get what you can survive on, instead of getting healthy things because they cost more!*”

—Participant 5, Female

This issue forces individuals to resort to less healthy options or to find alternative sources of food, such as Kraft dinners or frozen or canned fruits and vegetables, that do not align with traditional dietary practices and may lack the cultural significance of traditional foods:

“*I can’t obviously afford all those prices all the time. So, then it either reverts me to other sources, or I must look to frozen or canned fruits and vegetables and lentils, etc.*”

—Participant 2, Female

“*Personally like, I used to have a very strict vegetarian diet. So I was always a little bit health conscious, but as things have got like the cost of food has risen. So I’m a little bit more selective about what I can buy. But sometimes I have to buy the easy things, … like Kraft dinner.*”

—Participant 3, Female

The following quote from Participant 4 underscores the trade-offs many are forced to make between affordability and nutritional quality, highlighting a critical barrier to healthy eating that stems from economic limitations and poverty:

“*Biggest reason why we go to the store is because it’s cheap and it allows us to keep like our children fed like, even though we’re not the happiest about having to eat that type of food. Sometimes …you have to do what you have to do to survive. If there was an alternative option, we would take it.*”

—Participant 4, Female

The financial burden is further exacerbated for those with specific dietary needs which require more expensive specialized products:

“*…gluten makes me sick and it’s hard because everything has gluten, and the things that don’t have gluten cost so much more money.*”

—Participant 4, Female

“*First Nations are lactose intolerant, we only buy lactose-free milk. A jug of that is more than double the price of a regular jug of 2% milk.*”

—Participant 12, Male

##### High Cost of Living

The high cost of living, particularly in urban areas, exacerbates the difficulty of affording healthy food. This challenge is compounded by the need to cover other essential expenses, leaving less room in the budget for quality food:

“*…the high cost of living, …that’s a barrier* [to healthy eating]*. What it costs to maintain housing seems like it just keeps getting bigger and bigger, and it’s getting harder to independently run a household with a small family*”

—Participant 4, Female

“*In urban centers people have increase access* [to healthy food] *but because of the high cost of living they can’t actually obtain what they need.*”

—Participant 7, Female

##### Economic Constraints

Economic constraints, including limited income and reliance on social assistance, significantly limit the ability to purchase healthy foods. This issue is often more severe for urban Indigenous populations, who may also face additional challenges in accessing traditional nutrients:

“*Not many people get help from the reserves as an urban First Nations person. …social assistance with the Saskatchewan Government… only pays the bare minimum of either you are paying rent and bills, or you’re buying healthy food. You can’t get it all.*”

—Participant 5, Female

“*A lot of the barrier is income …depending on what their income situation is.*”

—Participant 8, Female

Participants also discuss how poverty, homelessness, and addiction further complicate access to nutritious food:

“*We have a lot of poverty, a lot of homelessness, and a lot of addiction. So people aren’t eating healthy…with the poverty people are not eating healthy. A lot more people are out on the streets, too, asking for money to eat.*”

—Participant 6, Female

#### 3.2.2. Theme 2: Access and Availability Issues

This theme explores the barriers to healthy eating stemming from the challenges in accessing traditional, organic, and fresh foods.

##### Limited Access to Traditional and Fresh Foods

Participants describe significant limitations in accessing both traditional foods and fresh produce, attributing this scarcity to a range of factors, including the urban food environment and seasonal availability:

“*People who are Indigenous and who are urban live under that poverty line, and unfortunately they don’t have access to that type of nutrients* [from traditional foods]”

—Participant 1, Female

“*…there’s the Saskatoon Food Bank as well, but fresh fruits and vegetables are hard to come by in the winter.*”

—Participant 9, Male

##### Geographical and Logistical Barriers

One significant challenge highlighted by participants is the difficulty in finding space to grow one’s own food in urban settings. This issue reflects a broader problem of access to land for gardening, which is crucial for those looking to supplement their diets with fresh produce:

“*In urban areas, you’re not obviously going to have space to …grow your own garden.*”

—Participant 2, Female

“*Even just having enough garden space to grow their own stuff is limited.*”

—Participant 14, Male

Participants also face significant geographical and logistical barriers when it comes to hunting and gathering traditional foods. These challenges are particularly acute for those living in urban areas or far from their ancestral lands, where such practices are integral to cultural and dietary habits:

“*A lot of people that live in urban areas have often moved away from their home communities. And so they’re not going to have access to a lot of those hunting and gathering methods.*”

—Participant 2, Female

“*And for us* [urban Indigenous people] *we’re abundant with that* [market foods] *we could just go to the store, but we can’t just go out and hop in a boat and go hunting.*”

—Participant 4, Female

##### Transportation Challenges

Transportation is identified as a significant barrier impacting not only access to stores but also to locations suitable for hunting and fishing, essential practices for accessing traditional foods:

“*My family, like my community, is roughly 600 km away by vehicle, and then, once you get there by vehicle, you have to go by boat to go hunting or fishing… So I think it’s hard for my family members as well to be able to travel through our own traditional territory, to hunt because of, like the rising cost of living and the cost of fuel, and what it takes to get there.*”

—Participant 4, Female

Participants also face difficulties with transportation for basic grocery shopping. This issue is particularly challenging for those without personal vehicles living in areas with inadequate public transportation options:

“*From the people I know, many sometimes don’t have a vehicle and transportation is difficult to get healthy foods.*”

—Participant 9, Male

“*I’ve got to take the bus to carry a bunch of groceries. That’s not easy to do every day or every week, even especially during the wintertime.*”

—Participant 11, Female

#### 3.2.3. Theme 3: Lack of Nutrition Education and Awareness

This theme underscores a critical barrier to healthy eating, highlighting the gap in knowledge and understanding regarding nutrition and healthy dietary practices among participants. This lack of education leads to misconceptions about food, hinders informed dietary choices, and often results in poor health outcomes.

Participants express a need for a shift in how urban Indigenous people view healthy eating, particularly within the constraints of a budget:

“*It’s gonna have to be a shift with urban aboriginal people in terms of what they consider healthy eating on a budget.*”

—Participant 1, Female

The importance of educating both children and parents on food security and healthy eating practices is highlighted as a fundamental step towards improving public health:

“*It’s important to teach children, especially about food security and teach parents on how to eat healthy on a budget. …really important things for food security and for people’s health.*”

—Participant 1, Female

This education is crucial in combating obesity and promoting a basic understanding of nutritional needs:

“*So you’ll see a lot of people that are obese because they don’t know how to properly eat*”

—Participant 1, Female

Further, even when individuals attempt to make healthier choices, they often find nutritional labels confusing and intimidating:

“*I feel confident in my ability to read the labels, but not always in understanding what’s on the labels like there’s so many huge words and ingredients that you just don’t understand and it’s alarming, because it’s like this can’t be good for my health.*”

—Participant 4, Female

The influence of childhood experiences on adult eating habits is also emphasized, pointing to the long-term impact of early access to healthy foods and nutritional education:

“*And basically, not having that* [healthy food consumption] *from childhood because you pick up those practices in childhood of what you eat. So, if they didn’t have access when they were young, they wouldn’t know* [how to eat healthy]”

—Participant 7, Female

#### 3.2.4. Theme 4: Urban Lifestyle and Technological Influences

This theme explores how modern living patterns, technology, marketing, and the challenges of time management and meal preparation impact dietary habits, often leading to less healthy food choices.

##### Sedentary and Fast-Paced Urban Living

Urban living, characterized by sedentary behaviors and a fast-paced lifestyle, presents significant challenges to maintaining a healthy diet:

“*People who are living in urban areas are very sedentary. We just stay at home.*”

—Participant 1, Female

The urgency of urban life often leads to reliance on quick, less nutritious food options:

“*That’s the disadvantage of the urban setting, I guess, it’s so much faster paced. You’re on the go constantly…So if I gotta work late, it really pushes us to the fast foods or the frozen foods because we have to be out the door in 30 min.*”

—Participant 12, Male

##### Influence of Technology and Unhealthy Lifestyle Choices

Technology plays a dual role, both as a facilitator of sedentary behavior and as a medium through which unhealthy food choices are promoted:

“*My girl…I have to get put her into programs in order to be healthy. So she’s in dance ballet, acting, volleyball, like she has to stay active. Otherwise, she will just stay home on the tablet and eat junk food, and unfortunately, that is where kids are going.*”

—Participant 1, Female

This trend towards technology-driven lifestyles contributes to unhealthy eating habits, particularly among children:

“*They are obese. They’re eating fruit loops just in front of a tube or a screen of some sort.*”

—Participant 1, Female

##### Marketing Influence

Marketing, especially marketing targeted at children, significantly influences food preferences and purchasing decisions, driving demand for less healthy food options:

“*The reason why you choose them is because I guess marketing. Kids see something on TV that they wanna try… You know they’re gonna ask their parents to buy it. So you’re gonna buy it.*”

—Participant 5, Female

##### Challenges of Time Management and Meal Preparation

The demanding nature of modern life, involving balancing work and family responsibilities, often results in limited time for meal preparation, pushing families towards quicker, less healthy options:

“*Just depending on kind of like my week, I work full time, and I also have 5 children…so sometimes it’s little bit more work to prepare a healthier meal like the carrots and veggies and cooking.*”

—Participant 6, Female

“*Time and money…So I think for me, I would love to serve healthy meals every day, but I don’t have the time to do that for my kids.*”

—Participant 10, Female

“*But because we’re so busy…a lot of times, it’s easier ordering takeout or buying frozen foods that just need to be warmed up.*”

—Participant 12, Male

#### 3.2.5. Theme 5: Governmental and Policy Barriers

This theme examines the regulatory and systemic challenges that exacerbate difficulties in accessing traditional foods and adequate nutritional support, particularly for urban Indigenous populations.

##### Regulations and Restrictions

Participants express frustrations with the regulations and restrictions that limit the availability of traditional foods. These rules can have profound implications for cultural practices and the ability to maintain a diet rooted in Indigenous heritage:

“*I used to serve traditional foods at the restaurant that I used to run…and there were certain things, of course, that you can’t serve because Canadian food regulations do not allow it. They don’t allow for me to sell moose meat or serve moose meat.*”

—Participant 1, Female

##### Inequity in Support for Urban Indigenous Populations

A notable disparity in support and resources for urban Indigenous individuals compared to those living on reserves is highlighted. Participants note a lack of community support and resources, which significantly impacts their food security:

“*We just don’t have the same access to resources as an urban member, as same as a treaty person would have moving on reserve.*”

—Participant 4, Female

Urban Indigenous individuals often feel neglected by municipal bodies and their own First Nations agencies and communities, highlighting a stark difference in community support compared to those living on reserves:

“*Here* [in the cities] *we just don’t have that same level of support from our membership and our administration as we would if we were living back home*”

—Participant 4, Female

“*It’s coming from kind of like municipal bodies versus coming from our own First Nations agencies. …being an urban member, it’s like you get forgotten and fall through the cracks sometimes and …then you lose that sense of community, and the community taking care of one another, and the Collectivism that happens in the North, you don’t really see it as much up here.*”

—Participant 4, Female

Participants also noted a perception that reserves often prioritize support for those living within their boundaries, thereby creating a gap in assistance for members residing in urban areas:

“*And a lot of the reserves don’t, I’m not gonna say help, but they don’t supplement the urban living Indigenous people like their members. They’ll help more the ones that are living on reserve as opposed to the ones living off reserve.*”

—Participant 8, Female

#### 3.2.6. Theme 6: Cultural and Community Factors

This theme investigates how the erosion of traditional knowledge and skills, along with the weakening of community connections and generational shifts in food preferences, pose significant challenges to maintaining healthy dietary practices.

##### Loss of Traditional Knowledge and Skills

Participants highlighted a disconnection from traditional dietary practices, often resulting from the loss of knowledge transmission between generations:

“*Unfortunately, because First Nations people are not used to eating that type of* [healthy, nutritious] *food… oftentimes cooks will only cook like fries, or chicken nuggets, or really not healthy food, no veggies, no fruit in their diet*”

—Participant 1, Female

“*And then, like berries and medicine, that knowledge isn’t always passed down. So some people won’t have that same access to traditional foods and try them.*”

—Participant 11, Female

##### Loss of Community Connection and Generational/Cultural Shifts in Food Preferences

The weakening of community bonds and changes in food preferences across generations contribute to a decline in the consumption of traditional foods:

“*You lose that connection you have to your people… a lot of our norms and cultures and traditions it’s based around food and eating.*”

—Participant 4, Female

This disconnection influences younger generations’ attitudes toward traditional foods:

“*I feel like younger generations now are more hesitant to eat* [traditional foods].”

—Participant 4, Female

Participants observe a marked difference in dietary choices between their generation and those of their parents or grandparents, often noting a decline in the consumption of traditional foods:

“*I think not my generation, but probably my mother’s and my deceased grandparents their foods choices probably were way healthier… But me as an urban First Nation, I didn’t grow up on that type of food. So I don’t like it, and neither do my children.*”

—Participant 6, Female

#### 3.2.7. Theme 7: Psychological Effects of Historical Trauma

Historical trauma, particularly the legacy of residential schools, has lasting effects on mental health and behaviors surrounding food. This trauma is not only a backdrop but an active influence on daily behaviors and attitudes toward nutrition. Participants highlight how experiences from these institutions affect their current relationship with food, often manifesting as mental health issues and altered consumption patterns.

“*We also have to keep in mind the residential school syndrome. Right?… When you have that type of mentality from residential schools, it brings about mental health issues and with food in terms of like hoarding food.*”

—Participant 1, Female

Further elucidating this point, the same participant reflects on the historical context and its nutritional implications:

“*Our ancestors and family members that did go to residential school were often malnourished.*”

—Participant 1, Female

This additional statement provides a direct link to the generational impact of these schools, underscoring a historical pattern of nutritional deprivation. The psychological imprint of such experiences influences contemporary food behaviors, as past scarcity can impact current attitudes and behaviors around food security and consumption.

#### 3.2.8. Theme 8: Climate Change and Seasonal Variations

This theme highlights the significant impact that climate change and seasonal variations have on wildlife, agriculture, and, subsequently, the accessibility of traditional and nutritious foods. These environmental factors play a crucial role in determining the availability of resources necessary for maintaining a diet that is both healthy and culturally relevant. Participants note the adverse effects of climate change on natural food sources, which are vital for traditional diets:

“*If we have a really warm winter, you’re gonna have massive amounts of deer ticks and moose ticks.*”

—Participant 1, Female

This observation underscores the broader ecological imbalances resulting from climate change, which can lead to decreased wildlife populations and increased challenges in hunting practices. Seasonal changes inherently influence the availability of food, but when compounded with environmental disruptions, the impact becomes even more pronounced:

“*This last year. I’m not sure what was going on environmentally, but barely anybody caught any moose this year. So that’s kind of odd.*”

—Participant 3, Female

#### 3.2.9. Theme 9: Personal Dietary Preferences and Attitudes

This theme explores how individual tastes, familial traditions, and cultural practices significantly shape dietary choices, sometimes posing challenges to adopting healthier eating habits.

Participants discuss the tension between personal or familial food preferences and the knowledge of their health implications:

“*Things I know is high in salt, sulfites, or whatever but my girl loves it so.*”

—Participant 1, Female

This quote reflects a common dilemma: balancing the enjoyment of certain foods with awareness of their potential health impacts. Similarly, Participant 1 acknowledges occasional indulgence in fast food as part of allowing children to enjoy their youth despite personal reservations about its nutritional value:

“*That being said, kids have to have their youth as well. So going to McDonald’s is not something that I like, and we don’t do often, but kids need to live too.*”

—Participant 1, Female

Participants also discuss how preferences for certain traditional or comfort foods, deeply rooted in familial love and tradition, can complicate efforts to eat healthily:

“*My mom, she loves to put lard and salt on her Bannock. That’s just part of her traditional food, whereas I’ve always said to her you can eat that mom in moderation but you’re going to clog your arteries.*”

—Participant 6, Female

Further, some participants express a strong attachment to certain types of foods, indicating a preference for familiar comfort foods over healthier options:

“*I am just totally into meat and potatoes.*”

—Participant 7, Female

“*I don’t usually look for them* [healthy foods]*. I know where the fruits and the vegetables are, …I’m specific and touchy about my likes and dislikes.*”

—Participant 7, Female

**Figure 2:**
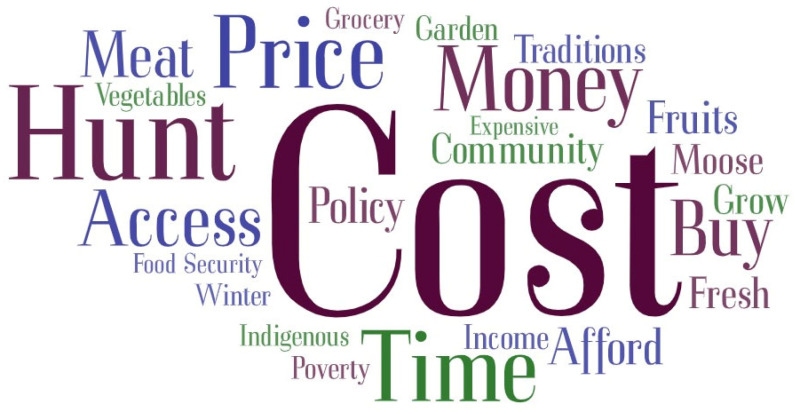
Word cloud of frequently used words in interviews on barriers to healthy eating.

A word cloud for the most frequently used words in the interviews on barriers to healthy eating is shown in Figure 2.

### 3.3. Facilitators to Healthy Eating

Seven major themes emerge from the thematic analysis of facilitators to healthy eating: (1) Community- and Family-Based Food Support Systems; (2) Connection to Traditional Practices and Knowledge; (3) Accessibility and Availability of Resources; (4) Health Consciousness; (5) Nutritional Knowledge and Educational Initiatives; (6) Self-Reliance and Skill Development; (7) and Economic and Employment Factors (Table 4).

#### 3.3.1. Theme 1: Community- and Family-Based Food Support Systems

This theme highlights the crucial role that family connections and community initiatives play in providing access to healthy and traditional foods, helping to overcome the challenges faced by urban and Indigenous populations.

##### Family and Community Engagement in Food Production

Deeply rooted in tradition and mutual support, families and communities play a crucial role in ensuring access to nutritious and culturally significant foods:

“*I know the value of going out, relying on family members, if need be, to go get your moose meat for the winter, to get your fish, to get your berries.*”

—Participant 1, Female

This reliance on traditional food sources, supported by familial and community networks, showcases a sustainable approach to food security and cultural preservation:

“*I also come from a family that my sister has* [significant gardening facilities]*, and so her food reaches all of us family members. I have tomatoes, cucumbers, berries, squash, beans, lettuce like just a whole ton of veggies for nothing* (no cost).”

—Participant 1, Female

“*I’m fortunate in the sense that we get food still shipped to me from my dad, who lives in the North and my brothers hunt when they can and my dad’s a fisherman.*”

—Participant 4, Female

##### Community Programs and Support Initiatives

Beyond familial networks, various community programs and support initiatives significantly contribute to improving food security and healthy eating among Indigenous and urban populations:

“*There’s been a lot more programs for indigenous people that makes healthy eating accessible. Whether it’s workshops, conferences or zoom calls that discuss the benefits of eating healthy.*”

—Participant 2, Female

“*One of the programs that really helped us recently was Jordan’s Principle. We’re lucky because we have our treaty right. So I guess that being treaty often helps us access things that other people can’t. So with Jordan’s Principle, we recently got approved for 3 months worth of groceries like they paid for $600 a month.*”

—Participant 4, Female

“*People living in urban areas have access to Food Bank that would be a facilitator and they have food markets. And they have different community organizations that can help out in a lot of areas.*”

—Participant 7, Female

Furthermore, during challenging times, such as the COVID-19 pandemic, community and programmatic support prove to be invaluable:

“*We were able to access a hamper with PAGC* (Prince Albert Grand Council) *during the* [COVID] *time which really did help.*”

—Participant 4, Female

#### 3.3.2. Theme 2: Connection to Traditional Practices and Knowledge

This theme underscores the importance of engaging with traditional food practices, cultural transmission of food preparation skills, and the communal sharing of food resources as vital components of healthy eating and cultural preservation.

##### Embracing Traditional Food Practices

Participants speak to the active efforts to maintain traditional food practices as an integral part of their lifestyle and identity:

“*I’m planning a hunting trip next weekend. …we’ll have some more wild meat in our fridge.*”

—Participant 14, Male

This forward-looking approach to hunting reflects a commitment to incorporating traditional foods into modern diets. The collaboration between community members and local farmers highlights an adaptive strategy to ensure access to traditional hunting grounds:

“*We have hunters on every reserve. And this is something that is just traditional knowledge, right?*”

—Participant 1, Female

“*Some of my brothers are lucky that they’ve developed relationships with farmers. So the farmers will call and allow them access to their land, and sometimes even let them know if there’s moose on the land and they’ll let them come hunt*.”

—Participant 4, Female

##### Inter-Generational Transfer of Cultural Food Preparation Skills

The transfer of food preparation skills from one generation to the next is highlighted as a critical aspect of maintaining cultural heritage and ensuring the continuation of healthy, traditional eating practices:

“*They teach about the values of, foraging and hunting and fishing and growing their gardens.*”

—Participant 1, Female

“*My kokum* (grandmother) *is… the one who taught me and my siblings how to prepare and to pack the meat and dress it and season it.*”

—Participant 3, Female

##### Community Sharing and Food Distribution

Community sharing and food distribution emerge as central themes, illustrating how traditional practices extend beyond individual or familial benefits to support wider community well-being:

“*So a lot of the people in the community are moose hunting, and then usually there’s like a small feast like families get together to eat the delicacies, and then the men provide meat to like single mother families or anybody that’s not able to hunt and being away from that environment. It just disconnects you from being able to do that. So not only like your community connection, but food security that your community can provide.*”

—Participant 4, Female

“*So in our culture, when the men hunt like they don’t only take care of their families like they take care of their siblings and their aunt, and so like they’re making sure that everybody gets fed*”

—Participant 4, Female

#### 3.3.3. Theme 3: Accessibility and Availability of Resources

This theme underscores the significant role that physical access to grocery stores, markets, and diverse food options plays in facilitating healthy eating. Additionally, it highlights how vehicle ownership and transportation are crucial in overcoming geographical barriers to access nutritious foods.

##### Accessibility to Grocery Stores and Markets

The availability of grocery stores and markets is critical, especially in urban settings, providing a variety of fresh foods and diverse food options. However, participants noted disparities in access, particularly affecting those in rural or reserve areas:

“*We often have grocery stores and lots of stocked shelves.*”

—Participant 2, Female

“*So, when you’re living in the city, I feel it’s easier compared to living on a reserve to get those healthier foods.*”

—Participant 9, Male

##### Availability of Diverse Food Options

Urban areas offer a wider variety of food options, from organic aisles in supermarkets to local farmers’ markets. However, cost remains a barrier:

“*…there’s tons of availability. I would say, the only thing that comes into barriers definitely for me is cost*.”

—Participant 2, Female

##### Vehicle Ownership and Access to Transportation

Having a vehicle significantly enhances one’s ability to access healthier food options and take advantage of deals across different stores:

“*…I have a vehicle, too, so I can go to Safeway and get a good deal. … I’m able to go to other places to get deals on healthy food.*”

—Participant 6, Female

#### 3.3.4. Theme 4: Health Consciousness

This theme highlights the increasing awareness and proactive actions individuals are taking concerning their health, particularly with respect to their diet. This heightened consciousness is often motivated by personal health conditions or a desire to prevent them from being exacerbated, as well as influences from childhood upbringing that emphasize healthy and traditional food practices.

##### Health Conditions as a Motivator

For many individuals, being diagnosed with a health condition serves as a crucial motivator for altering dietary habits and becoming more cautious about food choices:

“*I did get diabetes but I’m under control now. But that is one of the major factors that make me not have awful food like that. So I just can’t*”

—Participant 1, Female

This acknowledgment highlights how health challenges can lead to significant lifestyle changes, especially in regard to dietary habits. Other participants express a similar sentiment, emphasizing the importance of diet in preventing diseases prevalent in their communities:

“*Diabetes runs kind of rampant in my family, plus being First Nation like, I’m more at risk for that and I need to avoid things that have a lot of sugar and gluten and dyes*”

—Participant 4, Female

“*For First Nations, people, diabetes is one of the biggest things and heart diseases is one of the biggest things that we have as First Nations, people. So you’re trying to eat healthy to save yourself from going through that.*”

—Participant 5, Female

##### Influence of Childhood Upbringing

Childhood experiences and upbringing also play a significant role in shaping dietary habits and health consciousness. Participant 1 reflects on the importance of traditional practices learned in childhood:

“*So we’re very fortunate there to have the forest there and stuff, and so Fiddlehead picking every spring was really important. But I grew up that way and that’s how come I’m able to have this life and lifestyle.*”

—Participant 1, Female

This connection to traditional food-gathering and preparation practices from a young age fosters a deeper understanding and appreciation for healthy, culturally relevant foods. Participant 14 shares a similar perspective, emphasizing the intention to pass these values on to the next generation:

“*Processed food, …we still have it, but we rarely eat it, and we try to have more cultural food…, we try to raise our kids that way, so hopefully, they’ll grow that way. My son’s very interested in hunting, so I think he’ll be alright. He’ll learn how to hunt for his family.*”

—Participant 14, Male

#### 3.3.5. Theme 5: Nutritional Knowledge and Educational Initiatives

This theme emphasizes the critical role that both formal education and informal knowledge transmission play in fostering healthy eating habits. It explores how nutritional knowledge and awareness can significantly impact dietary choices, highlighting various sources of this knowledge, from professional training to family teachings and public education programs.

##### Nutritional Knowledge

A strong foundation in nutritional knowledge enables individuals to make informed decisions about their diets:

“*I know how to prepare them. And I’m actually a cook by trade. So I know all about the food handling stuff.*”

—Participant 3, Female

This professional expertise not only aids in food preparation but also ensures that meals are nutritious and safe to consume. Similarly, personal awareness of nutritional needs and how to meet them is vital:

“*I always consider if I’m meeting my nutrient requirements by eating this food or if this type of food is higher in calories than I need.*”

—Participant 9, Male

##### Nutritional Education and Awareness

The importance of early education on nutrition is underscored, with parents and educational programs playing a crucial role in instilling healthy eating habits from a young age:

“*Teaching youth very early about that… even my girl, who’s 11… I asked her, do you want me to drop you off a sandwich or anything, she’s like? No, I was like, well, do you want to take a juice, and she’s like Mom, sugar is not good for my vocal cords, and it’s not good for my teeth or my breath.*”

—Participant 1, Female

Educational initiatives, whether through schools, community programs, or prevention workers, are identified as key facilitators in promoting nutritional knowledge:

“*Facilitators could be prevention workers, let’s say, and holding a healthy eating on a budget class.*”

—Participant 1, Female

#### 3.3.6. Theme 6: Self-Reliance and Skill Development

This theme highlights the empowerment and independence achieved through home cooking, meal planning, home gardening, and local trading. These skills not only contribute to healthier eating habits but also foster a deeper connection to food and a sense of community.

##### Home Cooking and Meal Planning

Participants express a strong preference for preparing their own meals. This dedication extends to preserving foods and making condiments from scratch, illustrating a deep engagement with what they consume:

“*Canning season is coming up, and I have to think about my spaghetti sauce, my salsa, my pickled beets, and my pickles. I refuse to buy food that I can make.*”

—Participant 1, Female

“*I’ve been learning how to make …our own ketchup, …sauces for our pasta, and instead of buying it prepackaged just because it’s a little bit healthier and cost-effective.*”

—Participant 4, Female

Participants also share a sense of enjoyment and satisfaction derived from cooking, viewing it as a preferable alternative to relying on ready-made or processed foods:

“*So I get more fun out of it, like cooking chickens or stuff like that, instead of just buying the one that’s just readily available, and you can just go home and eat it. So I enjoy cooking my food better than something I would just throw in the oven and be done in like 20 min*.”

—Participant 13, Male

##### Home Gardening and Local Trading

Growing food and participating in local food exchanges are highlighted as extensions of the self-reliance fostered through cooking. These activities provide direct access to fresh, nutritious ingredients and strengthen community ties:

“*They teach about the values of, foraging and hunting and fishing and growing their gardens.*”

—Participant 1, Female

“*We had 2 gardens. We had chickens we always traded with the local farmers.*”

—Participant 1, Female

#### 3.3.7. Theme 7: Economic and Employment Factors

This theme addresses how income levels, economic stability, employment opportunities, and the ability to access sales and discounts play a pivotal role in influencing healthy eating habits. It underscores the interconnectedness of financial well-being with the ability to make healthier food choices.

##### Income Level and Economic Stability

Income level and overall economic stability are highlighted as key determinants of access to healthy food and the capacity to engage in activities like gardening, which can supplement dietary choices with fresh produce:

“*I would probably say, income level, access to healthy food, learning how to garden, maybe.*”

—Participant 7, Female

Participants noted that residing in urban areas often means higher income levels, which can offset the higher costs associated with purchasing healthy foods in city grocery stores:

“*Here in the city, we have more income because grocery stores are so expensive, and even like healthy foods.*”

—Participant 11, Female

##### Employment Opportunities

Employment opportunities in urban areas are seen as a facilitator for accessing healthy food due to the associated increase in income and economic stability:

“*There is more employment opportunity here for urban Indigenous people.*”

—Participant 4, Female

“*There are more amenities for Indigenous people within urban areas as opposed to on reserve. And that’s why a lot of people migrate to the cities, to the urban areas because of reasons like, education and employment.*”

—Participant 8, Female

##### Sales and Discounts

The strategic use of sales and discounts is identified as a critical strategy for making healthy eating more affordable, particularly in urban settings:

“*Things at the store that you keep watch of sales for healthy eating would be the only way you can afford things like that as being in the urban area.*”

—Participant 5, Female

“*We do stockpile when we can. We do have a deep freeze at home. But like I said, it’s more or less finding what’s on sale when we go to a supermarket. That’s a big thing, trying to save as much, stretch our dollar as far as we could.*”

—Participant 12, Male

**Figure 3:**
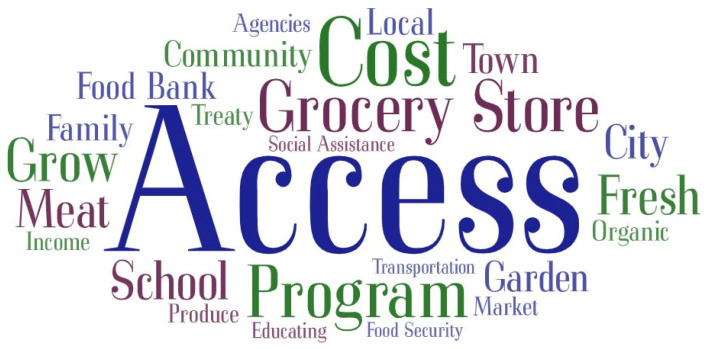
Word cloud of frequently used words in interviews on facilitators to healthy eating.

The word cloud for the most frequently used words in the interviews on facilitators to healthy eating is shown in Figure 3.

### 3.4. COVID-19 Pandemic and Eating Habits

Six major themes emerged from the thematic analysis of the negative impacts of the pandemic on the eating habits of urban Indigenous households: (1) Economic Impact and Loss of Income; (2) Inflation and Rising Food Costs; (3) Supply Chain Disruptions Affecting Food Availability; (4) Limited Access to Preferred Foods; (5) Increased Reliance on Convenience Food Options; and (6) Disruption of Traditional Food Practices.

#### 3.4.1. Theme 1: Economic Impact and Loss of Income

The COVID-19 pandemic has had profound economic impacts on urban Indigenous populations, significantly affecting their eating habits due to income instability. Participants described how the pandemic led to job losses, reliance on social assistance, and the need to adapt household budgets, which in turn influenced their ability to purchase nutritious food.

“*Lucky for me, during the pandemic and lockdown, I was still able to work. So I still had an income. My partner, he’s a school teacher, so he did not have an income. My sons, who live actually in the basement suite, they were receiving the COVID benefits. So they, of course, would pitch in for groceries.*”

—Participant 6, Female

#### 3.4.2. Theme 2: Inflation and Rising Food Costs

The COVID-19 pandemic has significantly exacerbated the issue of food inflation, impacting the eating habits of urban Indigenous peoples by making it more difficult to afford healthy and fresh food options. Participants expressed their concerns about the noticeable increase in the prices of essential food items, which strained their budgets further.

“*The cost of food went up after COVID happened. Like, I remember being able to get a lot of food with one $150. Now, I feel like I only get like 12 items at a store for that much.*”

—Participant 5, Female

#### 3.4.3. Theme 3: Supply Chain Disruptions Affecting Food Availability

The COVID-19 pandemic significantly disrupted supply chains, leading to noticeable shortages of essential food items in grocery stores, which added to the challenges faced by urban Indigenous communities in maintaining a healthy diet.

“*Then, you see things on the shelf that just aren’t available, which kind of adds to the stress… You couldn’t find yeast. So you couldn’t like bake your own bread at home, and things like that, because the shelves were always so empty.*”

—Participant 4, Female

“*There was a shortage of particular foods. Sometimes it was hard to find certain things at the grocery store like fresh vegetables.*”

—Participant 8, Female

#### 3.4.4. Theme 4: Limited Access to Preferred Foods

The COVID-19 pandemic greatly affected the dietary choices and access to preferred foods for urban Indigenous communities, influencing their ability to maintain a healthy diet with fresh produce.

“*It impacted our ability to get, like all the healthy food that I would usually stock up like every Sunday we buy the fruits and the veggies, and make sure like that’s always there for my son, like we weren’t able to do that for a long time, and we were dependent on a lot of like dry food like Pasta, and instant things.*”

—Participant 4, Female

#### 3.4.5. Theme 5: Increased Reliance on Convenience Food Options

The COVID-19 pandemic led to a shift towards convenience food options among urban Indigenous communities, as families faced the challenges of isolation and limited access to regular grocery shopping. This reliance on ready-to-eat meals from delivery services and processed foods highlights the strain on dietary habits during the pandemic:

“*When we did get sick we had to all isolate in a separate room… My son wasn’t sick and he had to be separate from us. And we had to basically get him like, skip the dishes every day for 10 days to prevent him catching our germs.*”

—Participant 4, Female

“*During the pandemic, my older daughters, who are 20, they utilized Skip the Dishes and that sort of stuff a lot more, for whatever reason.*”

—Participant 12, Male

#### 3.4.6. Theme 6: Disruption of Traditional Food Practices

The COVID-19 pandemic significantly disrupted traditional food practices among urban Indigenous communities, highlighting the broader impacts on cultural and communal activities that are integral to their way of life. These disruptions affected not just the physical act of gathering and hunting but also the communal aspects of sharing and preparing meals together:

“*And hunting was not like before. We couldn’t go and like, hang out with people and eat big meals anymore because of the COVID mask situation, all that stuff, and not being allowed in the household.*”

—Participant 13, Male

## 4. Discussion

In this qualitative study, we used inductive thematic analysis to examine the perceptions of healthy eating, along with barriers and facilitators, among urban Indigenous individuals living in Saskatchewan, Canada. Our research uncovers a complex web of experiences and insights, highlighting the intricate relationship between cultural traditions, socioeconomic factors, and modern urban challenges in shaping dietary habits. Participants express a strong preference for nutrient-rich, natural, and minimally processed foods, emphasizing the importance of incorporating traditional Indigenous foods into their diets. This preference is deeply tied to cultural identity, highlighting the intrinsic value of traditional foods not only for their nutritional benefits but also as a vital link to cultural heritage and community well-being. However, the pursuit of healthy eating is significantly challenged by a range of barriers, including economic constraints, limited access to traditional foods, and the psychological impacts of historical trauma. These barriers were notably intensified during the COVID-19 pandemic, which amplified issues such as economic instability, food inflation, and disruptions in food supply chains, further complicating access to nutritious and preferred foods. Despite these challenges, our study also identified critical facilitators to healthy eating, such as community and family support, engagement in traditional food practices, and a growing awareness of the importance of nutritional knowledge.

**Figure 4:**
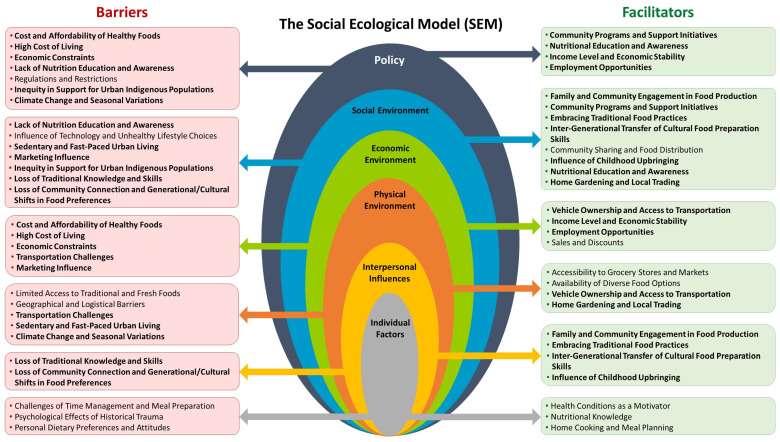
Conceptual framework of barriers and facilitators to healthy eating among urban indigenous populations based on the Social Ecological Model (SEM). The bolded items are those that are repeated across multiple layers.

The results from our study have led to the development of a conceptual framework enhanced with an intersectionality lens (Figure 4) based on the SEM. This framework focuses on different levels of the SEM—individual factors, interpersonal influences, physical environment, economic environment, social environment, and policy.

Our study reveals that urban Indigenous populations perceive healthy eating as a blend of mainstream nutritional advice—which emphasizes nutrient-rich foods, minimizes unhealthy ingredients, focuses on home-cooked meals, and adheres to dietary guidelines—and a deep respect for traditional foods. This approach demonstrates the integration of modern nutritional science while still respecting ancient dietary wisdom. These findings are echoed and expanded on by other research, creating a detailed narrative about the dietary preferences and challenges faced by Indigenous populations in various contexts. For example, Buksh et al.’s study on urban Indigenous Fijian mothers’ perceptions similarly emphasized the importance of traditional cuisine and food preparation methods in regard to healthy eating [30]. This study underscored the complex, multifaceted nature of healthy eating within Indigenous communities, where traditional foods are not only preferred for their freshness and natural qualities but also act as crucial elements in preserving and transmitting culture [30]. Furthermore, the emphasis on maintaining a balanced diet, incorporating traditional cuisine and methods while navigating modern dietary challenges, is a theme that resonates deeply with our findings. This balance involves not just a selection of what to eat—focusing on traditional and locally grown foods over processed options—but also how to eat, such as managing portion sizes and integrating foods from all food groups to achieve a balanced diet [30]. Similarly, Goettke and Reynolds’ study on the Cree Nation of Nemaska offered valuable insights into the complex interplay between traditional and modern dietary practices among Indigenous communities [18]. This study revealed how Indigenous communities value traditional foods and practices as core elements of their cultural heritage while adapting to lifestyle changes and external influences. The emphasis on traditional foods as being inherently healthy, combined with adaptive strategies to incorporate modern cooking methods, illustrates a dynamic blend of ancestral wisdom and contemporary nutritional knowledge [18]. This dichotomy illustrates the ongoing negotiation within Indigenous communities between maintaining traditional foodways and adapting to changes in lifestyle and environment brought on by external influences.

Traditional foods, recognized as being integral to the health and cultural heritage of Indigenous peoples, are associated with improved dietary quality, as supported by the existing literature [31,32]. However, urban Indigenous populations face a complex array of barriers to accessing traditional foods [17,33,34], as highlighted in our findings and illustrated in the conceptual framework. At the individual level, challenges include time constraints and a lack of knowledge about preparation techniques. Interpersonally, the erosion of traditional food preparation skills and loss of intergenerational knowledge transfer further impede access. At the physical environment level, difficulties in accessing land and natural resources are significant impediments, while climate change poses an external threat to food availability, complicating efforts to harvest and gather traditional foods. The economic environment exacerbates these issues, with high costs associated with acquiring traditional foods and limited financial resources further constraining access. The social environment also plays a crucial role, as urban settings often lack the community and cultural support systems that facilitate traditional food practices. This reliance on social networks for access to traditional foods highlights the importance of maintaining cultural and kinship ties even in urban environments. Lastly, at the policy level, government regulations and restrictions on fishing and hunting rights, along with broader environmental changes, compound these barriers, making traditional foods less accessible and affordable for urban Indigenous populations (Figure 4). These challenges align with the literature, which underscores the complex nature of accessing traditional foods within urban settings. Our previous research revealed that over half of the urban Indigenous population in Saskatchewan (54.2%) reported insufficient time to engage in hunting, fishing, foraging, or preparing traditional foods, which highlights a major conflict between urban lifestyles and the time-consuming nature of traditional food practices [17]. Furthermore, nearly 38% of participants reported difficulties in accessing traditional foods due to urban environments lacking necessary natural resources and space. An equal percentage indicated a gap in knowledge about how to utilize, prepare, or obtain traditional foods, signifying a loss in the transmission of vital cultural culinary knowledge [17]. These barriers are compounded by broader issues such as climate change affecting traditional food harvesting and consumption (38.9%) and financial costs for acquiring traditional foods (36.7%), which resonate with the challenges outlined by Cidro et al. [33] and Elliott et al. [34]. Cidro et al. underscored the importance of connection to land and community in accessing traditional foods, noting that urban Indigenous peoples often rely on being “gifted” food from relatives or engaging in bartering practices [33]. This reliance on social networks for access to traditional foods highlights the significance of maintaining cultural and kinship ties even in urban environments. Elliott et al. further elaborated on the multifarious factors that limit access to traditional foods in urban centers [34]. These included disconnection from family and home communities, a shift towards a consumer mentality, and the loss of traditional knowledge due to the lasting impacts of colonization and residential schools. Additionally, government policies that restrict fishing and hunting rights, along with environmental changes, compound these barriers, making traditional foods less accessible and affordable for urban Indigenous populations [34]. These findings, along with our own, underscore a critical theme echoed in our research: the urban environment poses unique challenges to accessing traditional foods, from the logistical difficulties of hunting and gathering in an urban context to the socio-economic barriers of high living costs and loss of community support.

In light of the financial barriers to healthy eating identified among Indigenous populations in urban areas, our study, alongside the existing literature, offers comprehensive insights into the multifaceted challenges impacting these communities [35,36,37]. Our findings highlight how the affordability of healthy foods, economic hardships, and the high cost of living significantly influence the dietary choices of urban Indigenous adults. At the economic environment level, high costs and economic constraints significantly impact individuals’ ability to purchase healthy foods, often forcing compromises in dietary quality and leading to a reliance on less nutritious options. Additionally, the high cost of living further exacerbates these challenges by reducing the disposable income available for food purchases. At the policy level, inadequate support for healthy eating initiatives and subsidies favoring unhealthy food options further exacerbate these barriers. Government regulations and lack of financial assistance programs specifically targeting the affordability of healthy foods for urban Indigenous populations limit their access to nutritious food options. This is consistent with Abbott et al.’s study on the economic constraints that hinder participants’ access to healthy foods, where high food costs force compromises in dietary quality, leading to increased dependence on less nutritious options [35]. Similarly, Kerpan et al. identified income as a crucial barrier to healthy food purchasing among urban Indigenous youth, emphasizing the role of economic limitations in shaping dietary behaviors from a young age [36]. Skinner et al. expand upon these themes within a broader context through their scoping review, which underscores the consistency of affordability and access challenges across diverse urban Indigenous communities in Canada, the United States, and Australia [37]. The review revealed a consistent theme across studies: the significant challenge posed by the affordability and access to store-bought, healthy foods for urban Indigenous populations. Like our study, the review detailed how market-based foods often remain out of reach for Indigenous individuals operating under tight budget constraints, with choices being heavily influenced by sale items to avoid the risk of food wastage [37]. This issue of affordability is not isolated but rather is intertwined with access challenges, where physical availability of stores, transportation limitations, and other financial commitments further complicate food security. In Canada and the United States, Indigenous peoples migrating from reserves to urban centers noted a comparative decrease in the cost of healthy foods, yet urban residents still faced significant barriers, such as higher prices in remote urban areas and the need to balance food purchases against other financial obligations [37]. Collectively, these studies highlight the complex interactions between economic factors, accessibility, and cultural considerations in shaping the food security landscape for Indigenous peoples in urban environments.

Our study highlights community- and family-based food support systems, the importance of connection to traditional practices and knowledge, and the critical role of accessibility to resources as key facilitators to healthy eating. As shown in Figure 4, at the individual level, personal health consciousness and knowledge about traditional and modern nutritional practices play a crucial role. Interpersonally, the support from family and community members in food production and preparation enhances dietary outcomes. Within the physical environment, the availability of community gardens and urban agriculture initiatives, such as Community Shared Agriculture (CSA) programs, provide accessible sources of traditional foods and foster food sovereignty, resonating with Cidro et al.’s findings [33]. The economic environment is positively influenced by employment opportunities and economic stability, which enable better access to healthy foods. Socially, community programs and support initiatives are essential in promoting nutritional education and awareness, as well as facilitating inter-generational transfer of cultural food preparation skills, as emphasized by Stotz et al. [38]. These initiatives underscore the diversity within American Indian and Alaska Native (AI/AN) communities and their effective diabetes nutrition education programs, which leverage the strength of community and family support systems, traditional foods, and traditional food acquisition and preparation practices [38]. At the policy level, the creation of culturally relevant food programs, such as Indigenous food banks focusing on cultural safety and the provision of traditional foods, as discussed by Richmond and Dokis [39], highlights the importance of supportive policies. This aligns with our study’s identification of community programs as being essential in facilitating access to healthy and traditional foods. However, concerns about the nutritional quality of provided foods in supplemental food programs, as noted by Dillinge et al. [40], highlight the need for healthier options and culturally sensitive nutritional counseling. Further, Gaudin et al.’s focus on Cree perspectives on traditional food consumption elucidates individual and community-level factors influencing dietary choices, aligning with our study’s emphasis on the multifaceted facilitators to healthy eating, from individual health consciousness to broader community and economic factors [41]. Lastly, Stotz et al. (2022)’s work on AI/AN serving organizations improving healthy food resources for older adults in urban areas further complements our findings [42]. The emphasis on community sharing, support, and the potential for organizational interventions to enhance food access and quality parallels our identified themes of community- and family-based food support systems, highlighting the potential impact of targeted, culturally sensitive programs on improving dietary outcomes [42]. These studies collectively underscore the multifaceted nature of facilitators to healthy eating among urban Indigenous populations, highlighting the pivotal role of community engagement, traditional practices, and supportive policies in enhancing nutritional health and food sovereignty. 

Implications: The findings of this study have important implications for public health policy, community programming, and nutritional education tailored to urban Indigenous populations. First, the emphasis on traditional foods and practices as a core component of healthy eating among study participants underscores the need for health initiatives that foster the reclamation and revitalization of Indigenous food systems [43,44]. At the individual and interpersonal levels, this could involve supporting urban Indigenous communities in establishing community gardens and green spaces dedicated to growing traditional plants and medicinal herbs [45]. Evidence suggests that the restoration of traditional food systems, along with the integration of healthy foods from contemporary diets, can significantly enrich nutritional diversity and profoundly improve nutritionally linked health outcomes [6]. Moreover, creating programs that facilitate access to traditional hunting and fishing territories, along with workshops on traditional food preparation and preservation techniques, can help bridge the gap between urban living and traditional practices. Second, the economic barriers to healthy eating identified in the study call for policy interventions that address food insecurity and affordability at the policy and economic environment levels. This might include advocating for policy changes that increase the availability of financial resources, such as food vouchers or subsidies specifically for purchasing healthy, traditional foods [46]. Third, the study highlights a critical need for culturally tailored nutritional education that respects and integrates Indigenous knowledge and perspectives at the social environment level. Educational initiatives should be co-developed with Indigenous communities, ensuring they are reflective of and relevant to the specific cultural practices, languages, and values of the community. This could take the form of culturally relevant nutrition guides, cooking classes that feature traditional foods, and school-based programs that educate Indigenous youth about the nutritional, cultural, and environmental aspects of their food. Fourth, acknowledging the significant impact of historical trauma and socioeconomic factors on health behaviors, health promotion efforts must adopt a holistic approach at all levels of the SEM [47]. This involves not only addressing the physical aspects of health but also supporting mental and spiritual well-being. Fifth, fostering strong partnerships between Indigenous communities, public health officials, policymakers, and researchers is essential at the policy and community levels to develop and implement strategies that address the unique challenges faced by urban Indigenous populations. Such partnerships could facilitate the creation of supportive environments that empower individuals to make healthy dietary choices, ultimately contributing to the reduction in health disparities and the promotion of wellness within these communities. Finally, the COVID-19 pandemic has underscored the necessity for robust, adaptable public health responses that can quickly address the exacerbation of existing barriers to healthy eating during health crises. This includes ensuring continuous access to affordable and nutritious food options, even amidst supply chain disruptions and economic downturns.

Strengths and limitations: This study possesses several notable strengths, including its qualitative design, which allows for an in-depth exploration of the perspectives and experiences of urban Indigenous individuals regarding healthy eating practices. The use of purposive and snowball sampling strategies facilitated the recruitment of a diverse participant group, enriching our understanding of the nuanced dietary habits and preferences within this community. The employment of inductive thematic analysis ensured that the themes emerged organically from the data, providing insights grounded in the participants’ lived experiences. Additionally, the application of the SEM enriched by the intersectionality lens as a theoretical framework enabled a comprehensive examination of the multiple layers of influence on healthy eating behaviors, ranging from individual factors to broader policy environments. However, the study also has limitations that warrant consideration. The geographical scope, limited to three major cities in Saskatchewan, may have restricted the generalizability of the findings to other urban Indigenous populations in Canada. The demographic composition of our sample, which was notable in that most participants had university degrees or were pursuing university degrees, presented another limitation. This educational background may have influenced dietary knowledge, attitudes, and practices, thus potentially not reflecting the broader experiences and challenges faced by the entire urban Indigenous population. Additionally, while the study aimed to capture a breadth of experiences, the voices of participants with limited access to technology or those less inclined to participate in research studies might have been underrepresented, possibly omitting critical perspectives on barriers to healthy eating.

## 5. Conclusions

In conclusion, our study provides valuable insights into the complex dietary practices and perceptions of healthy eating among urban Indigenous individuals in Saskatchewan, Canada. By employing a qualitative design, we have illuminated the profound influence of cultural traditions, socioeconomic challenges, and the importance of community support in shaping dietary habits within these communities. Our findings underscore the critical role of traditional foods and practices in not only nourishing the body but also in sustaining cultural identity and enhancing community well-being.

The study highlights significant barriers to healthy eating across multiple layers of the SEM, including economic constraints, limited access to traditional foods, and the lingering effects of historical trauma. At the policy level, inadequate support for healthy eating initiatives and unfavorable economic conditions further exacerbate these challenges. At the economic environment level, high living costs and limited financial resources significantly impede access to healthy foods. However, our study also identifies key facilitators that support healthy dietary choices across these layers. Community- and family-based food support systems, engagement in traditional practices, and increasing awareness of nutritional knowledge at the individual, interpersonal, and community levels play pivotal roles in promoting healthy eating. The availability of community programs and supportive policies at the societal level further enhances these facilitators. These insights have profound implications for public health policy, community programming, and nutritional education, pointing towards the need for culturally sensitive and community-engaged health promotion strategies. Policies and programs that support the reclamation and revitalization of traditional food systems, improve economic conditions, and foster strong community support networks are essential for improving the dietary health and overall well-being of urban Indigenous populations. Moreover, the lessons learned from the COVID-19 pandemic highlight the importance of incorporating pandemic preparedness into these strategies, ensuring that future crises do not exacerbate existing disparities in food security and health.

Future research should delve deeper into the interaction between urban development and traditional food access, investigating how urban planning and policy can be better aligned to support the dietary needs of Indigenous populations. Additionally, longitudinal studies could provide more detailed insights into the long-term impacts of integrating traditional foods and practices into the daily lives of urban Indigenous communities. Expanding research to include a wider variety of urban settings and Indigenous groups could further enrich our understanding of the diverse experiences and dietary strategies of these communities.

## Figures and Tables

**Table 1:** Participants’ characteristics.

	Mean ± SD (Range)
Age (Years)	39.8 ± 11.7 (21–61)
Annual Household Income (CAD)	CAD 72,600 ± CAD 30,692 (CAD 20,000–CAD 120,000)
	n (%)
Indigenous Identity	
First Nation	12 (85.8%)
Métis	1 (7.1%)
Unknown	1 (7.1%)
Place of Residence	
Saskatoon	7 (50.0%)
Regina	5 (35.7%)
Prince Albert	2 (14.3%)
Gender	
Female	10 (71.4%)
Male	4 (28.6%)
Education Level	
High School Education	3 (21.4%)
Undergraduate Education	8 (57.1%)
Graduate Education	1 (7.1%)
Non-degree Certification	1 (7.1%)
Professional Certification	1 (7.1%)
Employment Status	
Employed	8 (57.1%)
Unemployed	6 (42.9%)
Household Size	
1	2 (14.3%)
2	4 (28.6%)
3	2 (14.3%)
4	3 (21.4%)
≥5	3 (21.4%)
Home Ownership	
Own	2 (14.3%)
Rent	11 (78.6%)
Unknown	1 (7.1%)

**Table 2:** Themes and Subthemes for Perception of Healthy Eating.

Theme #	Themes	Subthemes
Theme 1	Focus on Nutrient-Rich Foods	Fruits and Vegetables
		Lean Proteins and Fish
		Nutrient-Dense Foods
Theme 2	Natural and Unprocessed Food Choices	Avoidance of Processed and Fast Foods
		Preference for Fresh and Natural Foods
Theme 3	Minimization of Unhealthy Foods and Ingredients	Reduction in Sugar and Salt
		Limited Intake of Starches and Carbs
		Limited intake of Red Meat
		Limited intake of Junk Food
Theme 4	Emphasis on Cultural and Traditional Foods	
Theme 5	Home-Cooked Meals and Cooking Practices	
Theme 6	Adherence to Dietary Guidelines	
Theme 7	Balanced and Diverse Diet	Well-Balanced Diet
		Moderation and Inclusion
Theme 8	Health-Adjusted Diet	

**Table 3:** Themes and Subthemes for Barriers to Healthy Eating.

Theme #	Themes	Subthemes
Theme 1	Economic Constraints and Poverty	Cost and Affordability of Healthy Foods
		High Cost of Living
		Economic Constraints
Theme 2	Access and Availability Issues	Limited Access to Traditional and Fresh Foods
		Geographical and Logistical Barriers
		Transportation Challenges
Theme 3	Lack of Nutrition Education and Awareness	
Theme 4	Urban Lifestyle and Technological Influences	Sedentary and Fast-Paced Urban Living
		Influence of Technology and Unhealthy Lifestyle Choices
		Marketing Influence
		Challenges of Time Management and Meal Preparation
Theme 5	Governmental and Policy Barriers	Regulations and Restrictions
		Inequity in Support for Urban Indigenous Populations
Theme 6	Cultural and Community Factors	Loss of Traditional Knowledge and Skills
		Loss of Community Connection and Generational/Cultural Shifts in Food Preferences
Theme 7	Psychological Effects of Historical Trauma	
Theme 8	Climate Change and Seasonal Variations	
Theme 9	Personal Dietary Preferences and Attitudes	

**Table 4:** Themes and Subthemes for Facilitators to Healthy Eating.

Theme #	Themes	Subthemes
Theme 1	Community- and Family-Based Food Support Systems	Family and Community Engagement in Food Production
		Community Programs and Support Initiatives
Theme 2	Connection to Traditional Practices and Knowledge	Embracing Traditional Food Practices
		Inter-Generational transfer of Cultural Food Preparation skills
		Community Sharing and Food Distribution
Theme 3	Accessibility and Availability of Resources	Accessibility to Grocery Stores and Markets
		Availability of Diverse Food Options
		Vehicle Ownership and Access to Transportation
Theme 4	Health Consciousness	Health Conditions as a Motivator
		Influence of Childhood Upbringing
Theme 5	Nutritional Knowledge and Educational Initiatives	Nutritional Knowledge
		Nutritional Education and Awareness
Theme 6	Self-Reliance and Skill Development	Home Cooking and Meal Planning
		Home Gardening and Local Trading
Theme 7	Economic and Employment Factors	Income Level and Economic Stability
		Employment Opportunities
		Sales and Discounts

## Data Availability

The data presented in this study are available on request from the corresponding author. The data are not publicly available due to privacy and ethical considerations related to working with Indigenous communities.

## References

[1] Government of Canada Indigenous Peoples and Communities. https://www.rcaanc-cirnac.gc.ca/eng/1100100013785/1529102490303.

[2] Statistics Canada How the Census Counts Indigenous People in Urban Areas. https://www150.statcan.gc.ca/n1/pub/11-627-m/11-627-m2022059-eng.htm.

[3] Saskatchewan Bureau of Statistics Indigenous Peoples of Saskatchewan. https://pubsaskdev.blob.core.windows.net/pubsask-prod/136766/2021%252BCensus%252BIndigenous%252Bpeoples.pdf.

[4] Hajizadeh M., Hu M., Bombay A., Asada Y. (2018). Socioeconomic inequalities in health among Indigenous peoples living off-reserve in Canada: Trends and determinants. Health Policy.

[5] Wilson K., Cardwell N. (2012). Urban Aboriginal health: Examining inequalities between Aboriginal and non-Aboriginal populations in Canada. Can. Geogr./Géogr. Can..

[6] Sarkar D., Walker-Swaney J., Shetty K. (2020). Food diversity and indigenous food systems to combat diet-linked chronic diseases. Curr. Dev. Nutr..

[7] Bodirsky M., Johnson J. (2008). Decolonizing diet: Healing by reclaiming traditional Indigenous foodways. Cuizine.

[8] King M., Smith A., Gracey M. (2009). Indigenous health part 2: The underlying causes of the health gap. Lancet.

[9] Wilk P., Maltby A., Cooke M. (2017). Residential schools and the effects on Indigenous health and well-being in Canada—A scoping review. Public Health Rev..

[10] Kim P.J. (2019). Social determinants of health inequities in Indigenous Canadians through a life course approach to colonialism and the residential school system. Health Equity.

[11] Skinner K., Hanning R.M., Desjardins E., Tsuji L.J. (2013). Giving voice to food insecurity in a remote indigenous community in subarctic Ontario, Canada: Traditional ways, ways to cope, ways forward. BMC Public Health.

[12] Keshavarz P., Lane G., Pahwa P., Lieffers J., Shafiee M., Finkas K., Desmarais M., Vatanparast H. (2023). Dietary Patterns of Off-Reserve Indigenous Peoples in Canada and Their Association with Chronic Conditions. Nutrients.

[13] Johnson-Down L.M., Egeland G.M. (2013). How is nutrition transition affecting dietary adequacy in Eeyouch (Cree) adults of Northern Quebec, Canada?. Appl. Physiol. Nutr. Metab..

[14] Damman S., Eide W.B., Kuhnlein H.V. (2008). Indigenous peoples’ nutrition transition in a right to food perspective. Food Policy.

[15] Power E.M. (2008). Conceptualizing food security for Aboriginal people in Canada. Can. J. Public Health.

[16] Kuhnlein H., Erasmus B., Creed-Kanashiro H., Englberger L., Okeke C., Turner N., Allen L., Bhattacharjee L. (2006). Indigenous peoples’ food systems for health: Finding interventions that work. Public Health Nutr..

[17] Shafiee M., Lane G., Szafron M., Hillier K., Pahwa P., Vatanparast H. (2023). Exploring the Implications of COVID-19 on Food Security and Coping Strategies among Urban Indigenous Peoples in Saskatchewan, Canada. Nutrients.

[18] Goettke E., Reynolds J. (2019). “It’s all interconnected… like a spider web”: A qualitative study of the meanings of food and healthy eating in an Indigenous community. Int. J. Circumpolar Health.

[19] Shafiee M., Keshavarz P., Lane G., Pahwa P., Szafron M., Jennings D., Vatanparast H. (2022). Food security status of indigenous peoples in Canada according to the 4 pillars of food security: A scoping review. Adv. Nutr..

[20] Tarasuk V., Mitchell A. (2020). Household Food Insecurity in Canada, 2017–2018.

[21] Statistics Canada Household Food Insecurity in Canada Early in the COVID-19 Pandemic. https://www150.statcan.gc.ca/n1/pub/82-003-x/2022002/article/00002-eng.htm.

[22] Pei C.S., Appannah G., Sulaiman N. (2018). Household food insecurity, diet quality, and weight status among indigenous women (Mah Meri) in Peninsular Malaysia. Nutr. Res. Pract..

[23] Huet C., Rosol R., Egeland G.M. (2012). The Prevalence of Food Insecurity Is High and the Diet Quality Poor in Inuit Communities. J. Nutr..

[24] Corbin J., Strauss A. (2014). Basics of Qualitative Research: Techniques and Procedures for Developing Grounded Theory.

[25] Thomas D.R. (2006). A general inductive approach for analyzing qualitative evaluation data. Am. J. Eval..

[26] Raine K.D. (2005). Determinants of healthy eating in Canada: An overview and synthesis. Can. J. Public Health.

[27] Tong A., Sainsbury P., Craig J. (2007). Consolidated criteria for reporting qualitative research (COREQ): A 32-item checklist for interviews and focus groups. Int. J. Qual. Health Care.

[28] Saunders B., Sim J., Kingstone T., Baker S., Waterfield J., Bartlam B., Burroughs H., Jinks C. (2018). Saturation in qualitative research: Exploring its conceptualization and operationalization. Qual. Quant..

[29] Anfara Jr V.A., Brown K.M., Mangione T.L. (2002). Qualitative analysis on stage: Making the research process more public. Educ. Res..

[30] Buksh S.M., Hay P., de Wit J.B. (2023). Perceptions on healthy eating impact the home food environment: A qualitative exploration of perceptions of indigenous food gatekeepers in urban Fiji. Nutrients.

[31] Sheehy T., Kolahdooz F., Schaefer S., Douglas D., Corriveau A., Sharma S. (2015). Traditional food patterns are associated with better diet quality and improved dietary adequacy in Aboriginal peoples in the Northwest Territories, Canada. J. Hum. Nutr. Diet..

[32] Blanchet R., Willows N., Johnson S., Salmon Reintroduction Initiatives O.N., Batal M. (2020). Traditional food, health, and diet quality in Syilx Okanagan adults in British Columbia, Canada. Nutrients.

[33] Cidro J., Adekunle B., Peters E., Martens T. (2015). Beyond food security: Understanding access to cultural food for urban Indigenous people in Winnipeg as Indigenous food sovereignty. Can. J. Urban Res..

[34] Elliott B., Jayatilaka D., Brown C., Varley L., Corbett K.K. (2012). “We are not being heard”: Aboriginal perspectives on traditional foods access and food security. J. Environ. Public Health.

[35] Abbott P., Davison J., Moore L., Rubinstein R. (2010). Barriers and enhancers to dietary behaviour change for Aboriginal people attending a diabetes cooking course. Health Promot. J. Aust..

[36] Kerpan S.T., Humbert M.L., Henry C.J. (2015). Determinants of diet for urban aboriginal youth: Implications for health promotion. Health Promot. Pract..

[37] Skinner K., Pratley E., Burnett K. (2016). Eating in the city: A review of the literature on food insecurity and Indigenous people living in urban spaces. Societies.

[38] Stotz S.A., Brega A.G., Gonzales K., Hebert L.E., Moore K.R., Group A.A.W.S. (2021). Facilitators and barriers to healthy eating among American Indian and Alaska native adults with type 2 diabetes: Stakeholder perspectives. Curr. Dev. Nutr..

[39] Richmond C., Dokis B. (2023). “We Make It Work Because We Must”: Narrating the Creation of an Urban Indigenous Food Bank in London, Ontario, Canada. Land.

[40] Dillinger T.L., Jett S.C., Macri M.J., Grivetti L.E. (1999). Feast or famine? Supplemental food programs and their impacts on two American Indian communities in California. Int. J. Food Sci. Nutr..

[41] Laberge Gaudin V., Receveur O., Girard F., Potvin L. (2015). Facilitators and barriers to traditional food consumption in the Cree community of Mistissini, Northern Quebec. Ecol. Food Nutr..

[42] Stotz S.A., Hebert L.E., Maddux A., Moore K.R. (2022). Healthy eating determinants and food security resource opportunities: Urban-dwelling American Indian and Alaska Native older adults perspectives. J. Nutr. Educ. Behav..

[43] Gendron F., Hancherow A., Norton A. (2017). Exploring and revitalizing Indigenous food networks in Saskatchewan, Canada, as a way to improve food security. Health Promot. Int..

[44] Joseph L., Turner N.J. (2020). “The old foods are the new foods!”: Erosion and revitalization of indigenous food systems in northwestern North America. Front. Sustain. Food Syst..

[45] Douglas V., Chan H.M., Wesche S., Dickson C., Kassi N., Netro L., Williams M. (2014). Reconciling traditional knowledge, food security, and climate change: Experience from Old Crow, YT, Canada. Prog. Community Health Partnersh. Res. Educ. Action.

[46] Lee A.J., Herron L.-M., Rainow S., Wells L., Kenny I., Kenny L., Wells I., Kavanagh M., Bryce S., Balmer L. (2024). Improving economic access to healthy diets in first nations communities in high-income, colonised countries: A systematic scoping review. Nutr. J..

[47] Joo-Castro L., Emerson A. (2021). Understanding historical trauma for the holistic care of indigenous populations: A scoping review. J. Holist. Nurs..

